# Surgical considerations for atrial functional regurgitation of the mitral and tricuspid valves based on the etiological mechanism

**DOI:** 10.1007/s11748-021-01629-x

**Published:** 2021-05-10

**Authors:** Toshihiko Shibata, Yosuke Takahashi, Hiromichi Fujii, Akimasa Morisaki, Yukio Abe

**Affiliations:** 1 Department of Cardiovascular Surgery, Osaka City University Postgraduate of Medicine, 1-4-3 Asahimachi, Abeno, Osaka 545-5956 Japan; 2 Department of Cardiology, Osaka City General Hospital, 2-13-22, Miyakojima Hondori, Miyakojima, Osaka 534-0021 Japan

**Keywords:** Atrial functional regurgitation, Valve repair, Surgical indication, Dual-valve disease

## Abstract

Atrial functional mitral regurgitation is a hot research topic in the field of mitral valve disease. Atrial functional mitral regurgitation is distinctly different from ventricular functional mitral regurgitation. The surgical indications for atrial functional mitral regurgitation have not been well established because of the small amount of evidence gathered to date. Mitral annular plication with an artificial ring is an essential surgical procedure because dilatation of the mitral valve annulus is a main factor underlying this pathology. Most of these cases can be treated by mitral annuloplasty alone. However, additional procedures, such as application of artificial chordae to the anterior leaflet for pseudo-prolapse, and posterior leaflet augmentation with a pericardial patch, are required in advanced cases with a giant left atrium and extremely enlarged mitral annulus. Chronic atrial fibrillation causes enlargement of the right and left atria. This pathology is a bilateral atrioventricular valve disease (dual-valve disease). Therefore, the conventional guidelines of single-valve disease should not be applied. Although atrial functional tricuspid regurgitation is underappreciated, tricuspid annuloplasty should be considered for most patients to prevent future regurgitation. In addition to the mitral and tricuspid valve procedure, integrated surgical management, including plication of the atrium and left appendage closure, is required. This review summarizes the current considerations of surgical treatment for atrial functional regurgitation of the mitral and tricuspid valves based on the etiological mechanism.

## Introduction

Mitral regurgitation (MR) has been broadly discussed as organic regurgitation and functional regurgitation. Organic regurgitation is caused by structural disorders of the leaflets and/or subvalvular apparatus, while functional regurgitation is caused by left ventricular dysfunction secondary to dilated cardiomyopathy and myocardial infarction. Therefore, the term “functional MR” is recognized as MR due to left ventricular dysfunction. Otsuji et al. [1] reported that significant MR is unlikely to occur with lone atrial fibrillation, and there has been no indication that atrial fibrillation itself can cause MR. Recent studies have indicated that significant MR occurs in some cases of chronic atrial fibrillation; this is referred to as atrial functional MR or atriogenic MR [2, 3]. Abe et al. [4] reported that 28% of patients with chronic atrial fibrillation lasting more than 10 years had significant MR and that 25% had significant tricuspid regurgitation (TR). Many reports have discussed the mechanisms of atrial functional MR using three-dimensional echocardiography [5–10].

The 2020 guideline for management of valvular heart disease in Japan [11] is the first to mention atrial functional MR among the world guidelines for heart valve disease. Since the concept of this disease has not yet taken root and is under-recognized worldwide, there is even less evidence for surgical treatment. The number of reports for atrial functional MR has increased in the last 2 years. This review summarizes the current status of surgical treatment of atrial functional regurgitation of the mitral and tricuspid valves based on the pathological mechanism.

## Annular dilatation of the mitral valve due to atrial fibrillation

Prolonged chronic atrial fibrillation causes dilatation of the atrium. The reason why the annulus of the mitral valve enlarges when the left atrium expands can be explained as follows. The mitral valve annulus is a structure of the left ventricle. In some patients, however, there is no continuity between the atrial and ventricular muscles (so-called disjunction), and only loose connective tissue connects them [12]. Konda et al. [13] reported that disjunction of the mitral valve was detected in 12% of patients without MR on echocardiography. Therefore, the mitral valve annulus, which is the base of the leaflet tissue, expands as the atrium expands. Annular dilatation results in shallow coaptation of the leaflets, resulting in MR.

## Definition of atrial functional MR

Atrial functional MR is generally defined as follows: (1) regurgitation associated with chronic atrial fibrillation, (2) left atrial enlargement and annular dilatation, (3) no significant organic change of the mitral valve apparatus, and (4) no impairment of left ventricular contractility [14]. Prolonged MR results in mild left ventricular enlargement and reduced contraction. Since various elements affect the mechanism underlying the development of MR, the definition of atrial functional MR has become ambiguous. The above-mentioned definition applies to patients in the early stage of atrial functional MR. The mechanism of MR is distinctly different from so-called functional regurgitation (ventricular functional MR) [14, 15].

## Characteristics of regurgitation in atrial functional MR

The rough zone of the mitral leaflet functions as the face of coaptation. Both leaflets push each other at the rough zone in the systolic phase to endure left ventricular pressure, and this might disperse stress on the chordae. When the annulus enlarges, the leaflets are pulled outward, the coaptation of the leaflets becomes shallower, and MR easily occurs. Some reports have indicated that the regurgitant jet of atrial functional MR in echocardiography flows straight forward [16, 17], whereas we often encounter an eccentric regurgitant jet that flows toward the posterior wall of the left atrium in atrial functional MR [14]. When the left atrial enlargement becomes prominent, the posterior mitral valve annulus is displaced outward, and the posterior mitral leaflet is pulled toward the left ventricle [18]; this is called “atriogenic tethering.” The movement of the posterior leaflet is restricted; therefore, the posterior leaflet is unable to play the role of a receiving partner of the anterior leaflet. Atriogenic tethering should be distinguished from traditional leaflet tethering seen in patients with ventricular functional MR due to ischemic heart disease or dilated cardiomyopathy. Atriogenic tethering and “hamstring leaflet” are similar, but the origins of the words and their meanings are slightly different. The word “tether” refers to a line or chain tying something down. In atrial functional MR, tethering represents a state in which the posterior leaflet is pulled toward the left ventricle by chordae (chain). As a result, the movement of the posterior leaflet is restricted. In contrast, the word “hamstring” may mean to disable or make powerless. “Hamstringing” was originally used to incapacitate a human or animal and render them incapable of effective movement by cutting the hamstring muscles. This word is also used as a metaphor of limiting something’s movement and usefulness. Tethering of the posterior leaflet disables the leaflet and prevents proper movement. Simply put, hamstring leaflet is a consequence of atriogenic tethering of the leaflet.

The hamstring sign occurs often in patients with a giant left atrium and extremely large mitral annulus and is sometimes seen in the early stages of disease. Therefore, hamstringing of the posterior leaflet is not always a determinant of atrial functional MR. Atriogenic leaflet tethering induces hamstringing of the posterior leaflet and further reduces leaflet coaptation, thereby worsening MR.

**Fig. 1:**
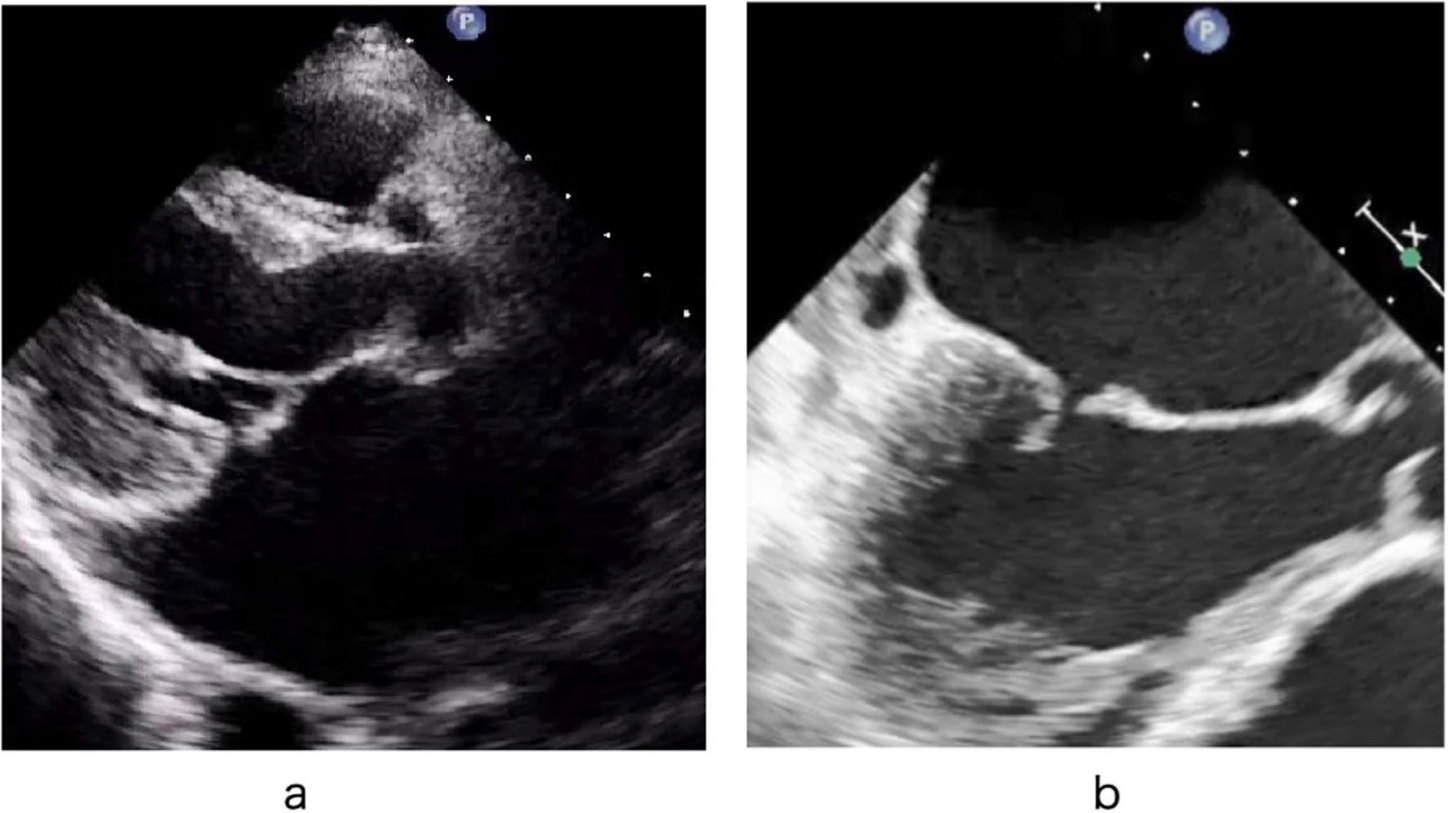
Pseudo-prolapse of the anterior leaflet. **a** Transthoracic echocardiography. **b** Transesophageal echocardiography

When the leaflet coaptation becomes shallow, the tip of the anterior leaflet rough zone sometimes shifts slightly toward the left atrium. This is called pseudo-prolapse or over-riding of the anterior cusp (Fig. 1). Pseudo-prolapse should be distinguished from type II MR. When the left atrium expands further, the mitral annulus expands markedly, creating a complete gap between the leaflets and leading to severe MR.

**Fig. 2:**
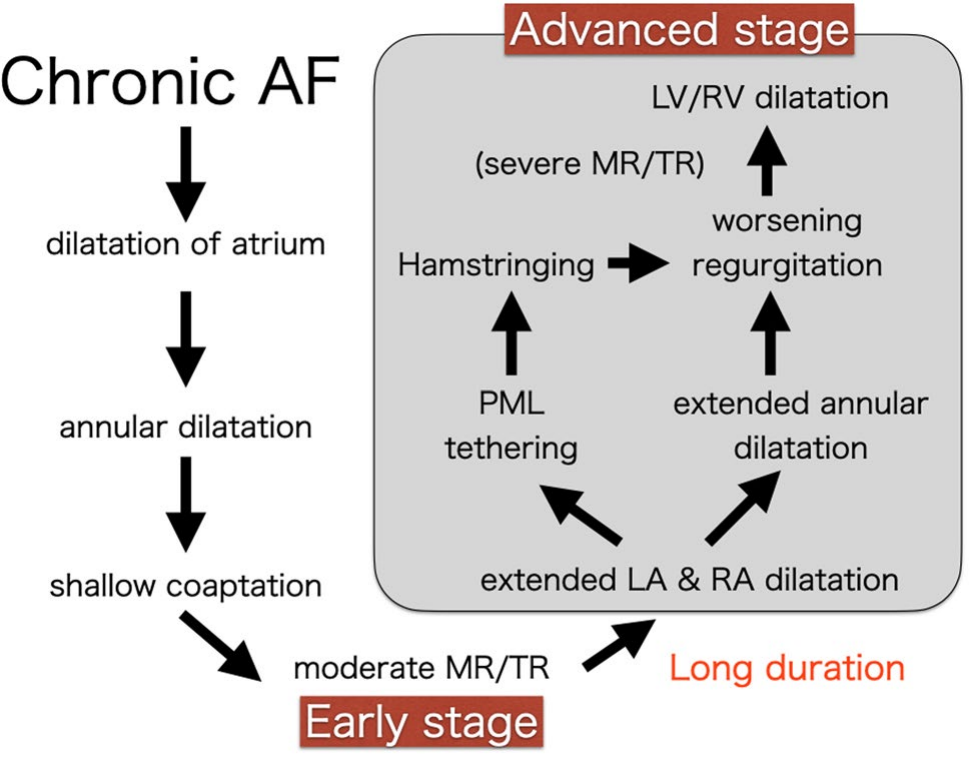
Progression of atrial functional MR. MR progresses through two stages of the early stage and the advanced stage

Prolonged severe regurgitation causes secondary left ventricular enlargement or left ventricular functional decline. This condition is often seen in patients who have atrial functional MR with a giant left atrium, and this situation is usually considered an advanced stage of atrial functional MR (Fig. 2).

These two stages have been discussed without differentiation, which makes understanding the pathology of atrial functional MR difficult. The advanced stage is multifactorial, and it is a complex pathological condition. The pathology of the advanced stage differs from that of the early stage, and the strategy for surgical treatment also differs.

## Surgical indications for atrial functional MR

The United States and European guidelines provide no indications for surgery for atrial functional MR. According to the 2020 Japanese guidelines for heart valve disease, mitral valve surgery is reasonable for patients with severe atrial functional MR who are persistently symptomatic, despite standard medical therapy for heart failure (Class IIa, Evidence Level C) (Table 1) [11]. The surgical indications for atrial functional MR are not yet well established because of the small amount of evidence.

**Table 1:** Treatment recommendations for atrial functional mitral regurgitation

Recommendation	COR	LOE
Standard medical therapy for heart failure, including diuretics, should be performed for symptomatic patients with atrial functional MR	I	C
AF catheter ablation is reasonable for symptomatic patients with persistent AF and severe atrial functional MR if successful ablation and maintenance of sinus rhythm can be expected from the duration of AF and the left atrial size	IIa	C
Mitral valve surgery is reasonable for patients with severe atrial functional MR who are consistently symptomatic despite standard medical therapy for heart failure	IIa	C

These recommendations can be applied to patients with chronic moderate atrial functional MR if the MR is severe with worsening heart failure or with exercise stress tests. Atrial functional MR is likely to accompany secondary atrial functional TR, and concomitant tricuspid surgery should be performed in patients with both atrial functional MR and TR. AF, atrial fibrillation; COR, class of recommendation; LOE, level of evidence; TR, tricuspid regurgitation. This table is taken from the 2020 guideline for management of valvular heart disease in Japan [11]

## Surgical procedures for atrial functional MR

Few surgical reports have focused on atrial functional MR. Mitral annular plication (MAP) with an artificial ring is an essential surgical procedure because dilatation of the mitral valve annulus is a main factor underlying this pathology. In 2010, Kilic et al. [19] reported a case of severe functional MR due to isolated annular dilation. In 2012, Vohra et al. [20] reported 20 surgical cases in which MAP was performed using a semi-rigid or rigid total ring. In 2015, we reported 10 surgical cases in which MAP for atrial functional MR reduced the MR severity, left atrial size, and recurrent heart failure [21].

Sakaguchi et al. [22] recently reported the surgical treatment of 20 patients with atrial functional MR. All patients underwent MAP, but four patients had recurrent MR and two of them required reoperation. One patient with recurrent MR had left ventricular enlargement and leaflet tethering. These authors stated that MAP alone is insufficient in some cases. We reported 45 surgical cases and demonstrated that the left atrial volume index was a predictor of postoperative cardiovascular events [23]. Recently, Balogh et al. reported a retrograde study of 131 patients who had surgery via mini-right thoracotomy [24], and the perioperative 30-day mortality was 0.1%. The patients had heart failure with preserved ejection fraction (HFpEF) and patients who had an annulus size > 32 mm were excluded in their study (median ring size was 28 mm). Therefore, we speculate that the mitral annulus was not extremely dilated in their study and the patients were not in the advanced stage, but still in the early stage, as mentioned above.

## Surgical strategies for pseudo-prolapse of the anterior leaflet

**Fig. 3:**
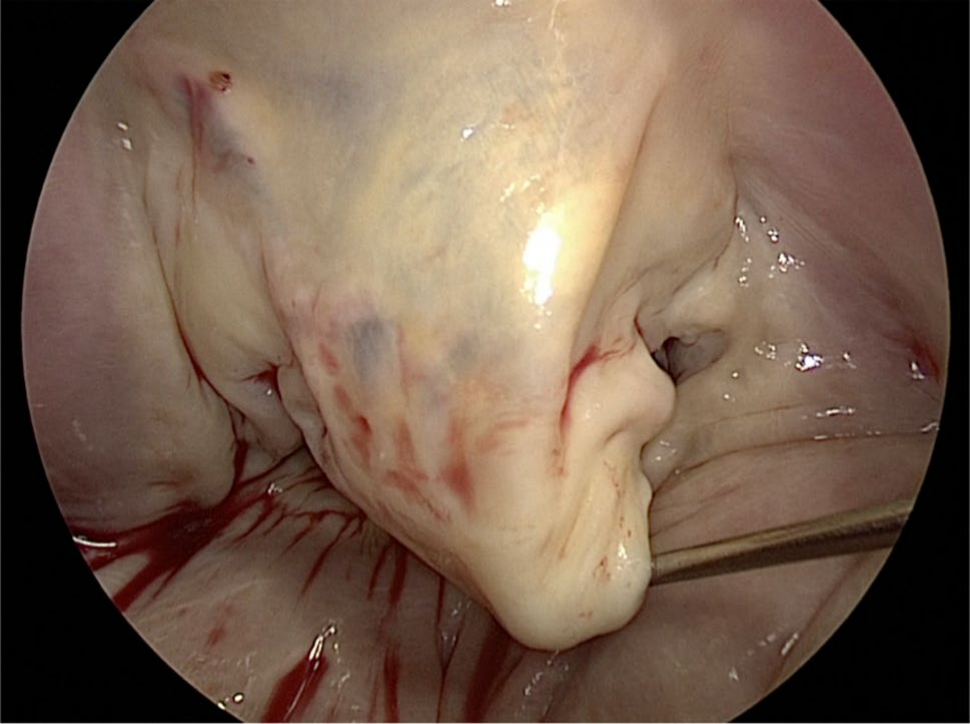
The rough zone of the anterior leaflet is slightly prolapsed in a patient with pseudo-prolapse

When pseudo-prolapse of the anterior leaflet is detected by echocardiography, slight elongation of the marginal chordae in the anterior leaflet is often observed during surgery (Fig. 3). Nazari et al. [25] described the stress distribution of the chordae. Normally, the marginal and secondary chordae share systolic stress. The thicker secondary chordae are exposed to greater stress, while the marginal chordae, which serve as the coaptation surface, are exposed to less stress. When the annulus is dilated, the coaptation surface becomes shallow, and the marginal chordae are loaded with the peak systolic stress.

Considering the above-described mechanism of stress distribution, we speculate that pseudo-prolapse of the anterior leaflet occurs as follows. When the shallow coaptation of the leaflet due to annular dilatation persists for a long time, the marginal chordae of the anterior leaflet may be exposed to overstress. Since the marginal chordae are much frailer than the secondary chordae, the marginal chordae might consequently be stretched and elongated. The secondary chordae, which are thicker than the marginal chordae, are not easily stretched. Therefore, the marginal chordae become elongated, and the rough zone of the anterior leaflet becomes slightly prolapsed (pseudo-prolapse).

Although MR with pseudo-prolapse of the anterior leaflet might be correctable using a small ring alone, no studies have provided any evidence to support this possibility. We added reinforcement of the rough zone by artificial chordae implantation using the loop technique to the pseudo-prolapsed area to avoid recurrent prolapse [23, 26]. Kaneyuki et al. reported mid-term results of mitral repair in 40 cases [27]. They applied artificial chordae replacement to the anterior leaflet for pseudo-prolapse in 19 (19%) patients, and 2 patients developed moderate to severe MR during follow-up. Further experience is required to determine whether artificial chordae are required in cases involving a pseudo-prolapsed anterior leaflet to achieve long-term durability.

## Operation for advanced cases with a giant left atrium

The mitral annulus is significantly enlarged in patients with a giant left atrium, and we use a larger ring in such cases [23]. We previously reported that patients with atrial functional MR often have a small posterior leaflet [21]. Therefore, in annuloplasty alone with an undersized small ring, achieving an appropriate coaptation area in patients with a small posterior leaflet may be difficult. There is a risk of ring dehiscence when the mitral annulus is over-shrunk using a ring that is too undersized. To solve these problems, we perform patch augmentation of the posterior leaflet with autologous pericardium to secure a sufficient coaptation surface with the posterior leaflet. We measure the length of the posterior leaflet, mainly the P2 area, with transesophageal echocardiography and intraoperative observation, and we perform patch augmentation in patients with a small posterior leaflet (height ≤ 10 mm) [23, 28]. This criterion is not strict and is determined by referring to the total annular size.

**Fig. 4:**
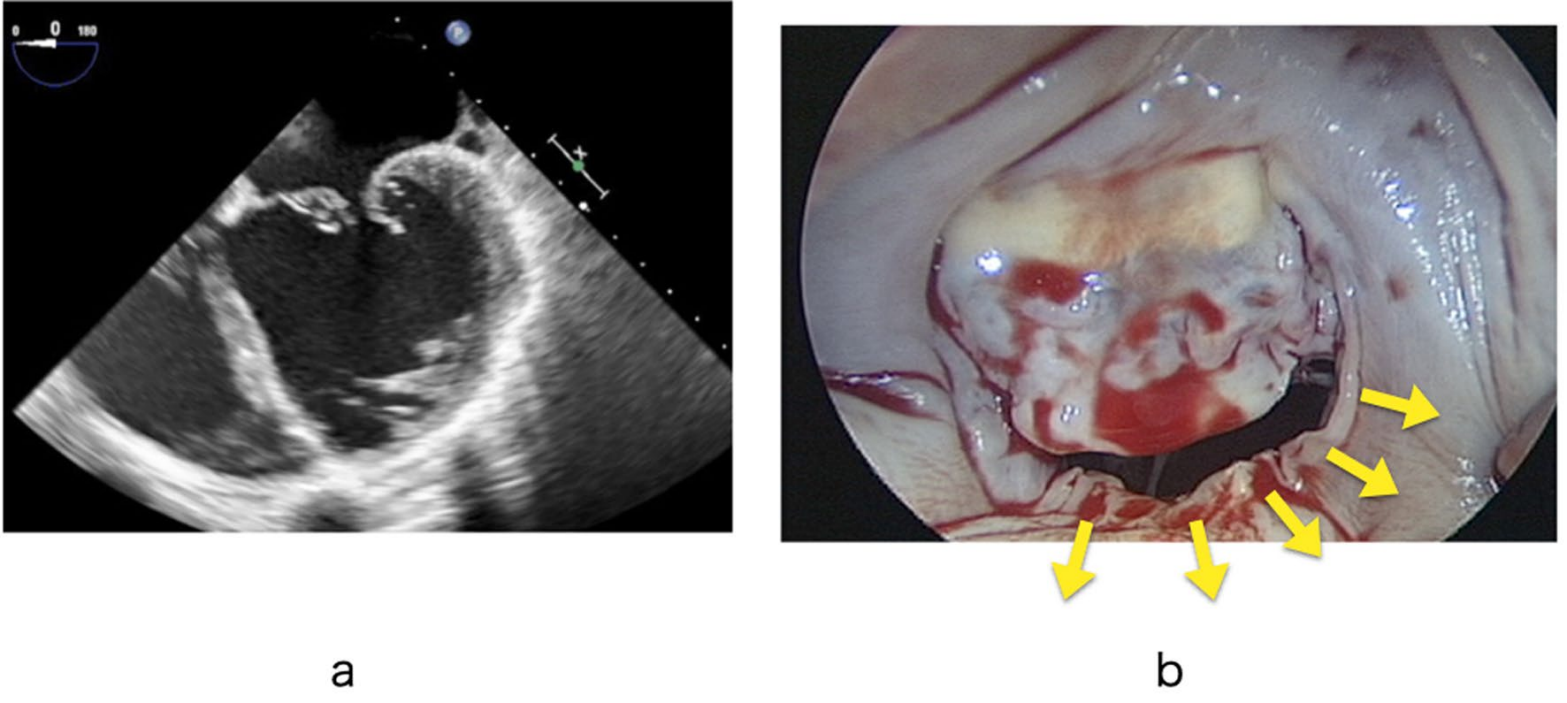
**a** Echocardiography of a patient with a giant left atrium showing a gap between the leaflets. **b** Photograph showing the large gap. The arrows show the direction of dilatation of the posterior annulus

**Fig. 5:**
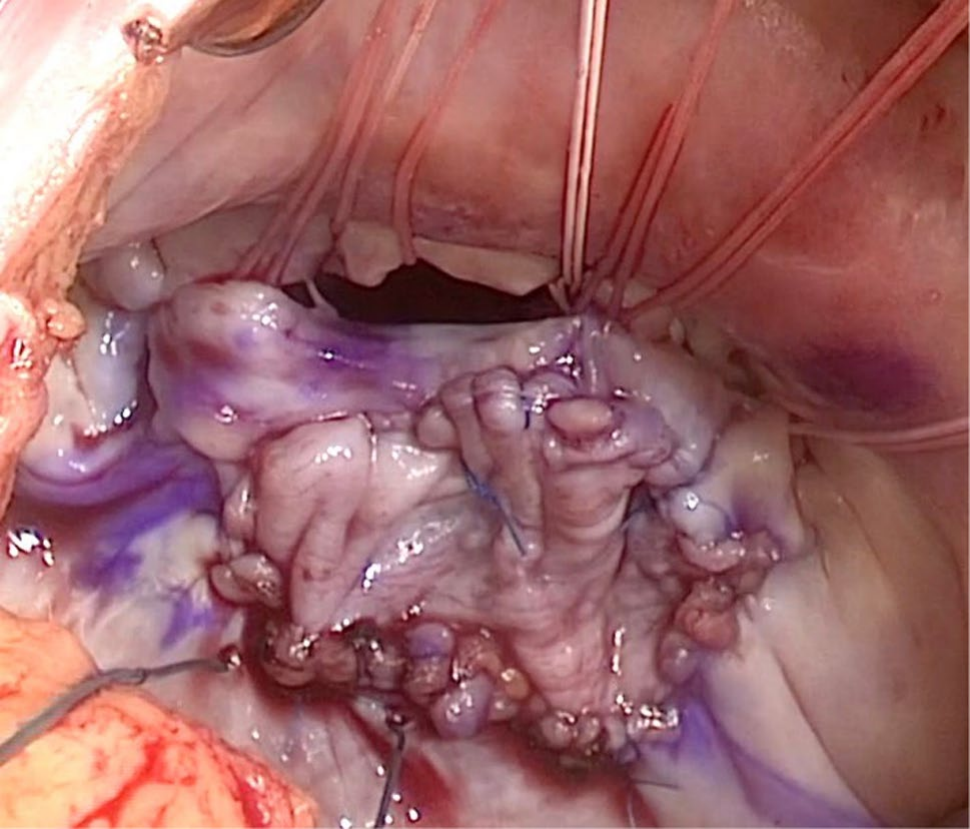
Patch augmentation of the posterior leaflet with an autologous pericardial patch

Annular enlargement mainly occurs in the posterior direction in advanced cases with a giant left atrium, and we have often observed a large gap between the leaflets in this area (Fig. 4). Kim et al. [29] reported that significant enlargement of the annulus occurs more often in the posterior annulus than in the anterior annulus. We apply patch augmentation mainly to P2, sometimes extending to P1 and P3. Importantly, the height of the patch should be large enough to ensure a sufficient final height of the posterior leaflet (Fig. 5).

Patch augmentation of the mitral valve with autologous pericardium has been applied for infective endocarditis, generative mitral disease, and rheumatic mitral disease. In addition, good long-term results with this procedure have been reported in some studies with or without glutaraldehyde treatment [30–32]. This procedure is controversial and challenging because of the risk of shrinkage, thickening, and calcification in the long term [33]. Most patients with a giant left atrium have a long history of atrial fibrillation and are older. We have applied patch augmentation in 16 advanced cases to date, and two cases required reoperation due to valve-related reasons (patch perforation and worsening regurgitation) (data not published). Therefore, mitral valve replacement with a bioprosthetic valve might be an alternative method to avoid a complicated and time-consuming technique using patch augmentation. Verifying the appropriate procedure for such advanced cases of atrial functional MR is necessary.

## Tricuspid valve repair as a treatment for dual-valve disease

Chronic atrial fibrillation causes enlargement of the right and left atria. Zhou et al. [34] reported that lone atrial fibrillation was associated with annular dilatation of the mitral and tricuspid valves and that annular dilatation and valvular regurgitation were significantly greater in the tricuspid valve. Silbiger [35] stated that atrial functional TR is underappreciated. The prevalence of significant TR was found to be 25% in patients with lone atrial fibrillation for longer than 10 years [4]. Which atrial enlargement is dominant (left or right) varies among individual patients, but surgical intervention for both atrioventricular valves should be required. Therefore, we use the term “atrial functional MR/TR” because regurgitation occurs in both atrioventricular valves.

MR and TR are significantly reduced by rest and administration of diuretics, and therefore, determining when the surgery should be performed is difficult [36]. If significant MR and TR remain after adequate medical treatment, there is a poor prognosis with a poor event-free rate [4]. The conventional guidelines for valvular disease describe the indications for surgical treatment of single-valve regurgitation. Notably, atrial functional MR/TR is a bilateral atrioventricular valve disease (dual-valve disease) [23]. Rather than the conventional idea of single-valve disease, regurgitation must be estimated as the sum of the regurgitation of both atrioventricular valves.

There have been no studies regarding surgical considerations for atrial functional TR. Silbiger showed that the patterns of right ventricular remodeling were different from atrial functional TR and ventricular functional TR (TR associated with left heart disease/pulmonary hypertension) and that leaflet tethering of the tricuspid valve was less than that of ventricular functional TR [35]. Therefore, TR in most patients should be able to be mostly treated by TAP alone. In our previous study, we added TAP to all surgical cases of atrial functional MR, even if TR was less than moderate to prevent regurgitation from worsening in the future [23]. Kaneyuki et al. reported that three patients developed moderate or severe recurrent TR during follow-up in five patients without TAP [27]. These authors suggested that TAP should be performed in addition to mitral valve repair of atrial functional MR. Balogh et al. added TAP in 67 (49%) patients, but they did not mention postoperative TR [24]. Whether some procedures, such as leaflet extension and neochordal implantation, are required in addition to TAP is still unknown. In our opinion, tricuspid annuloplasty should be performed simultaneously in all patients with atrial functional MR to prevent future regurgitation because atrial enlargement may continue as long as atrial fibrillation persists.

## Reduction of the atrial cavity size

**Fig. 6:**
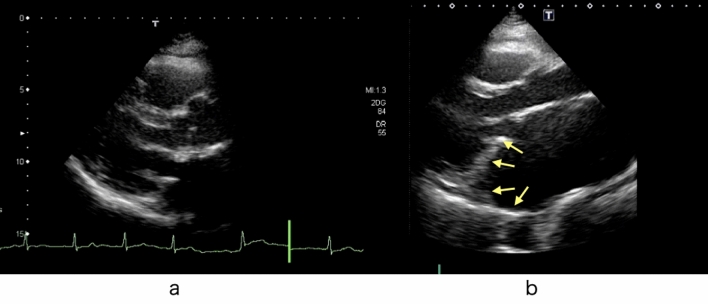
Postoperative echocardiography in a patient with atrial functional MR. **a** Immediately after the operation. **b** Twelve years after the operation. The left atrium was enlarged and the posterior mitral annulus was elevated by the enlarged atrial wall

**Fig. 7:**
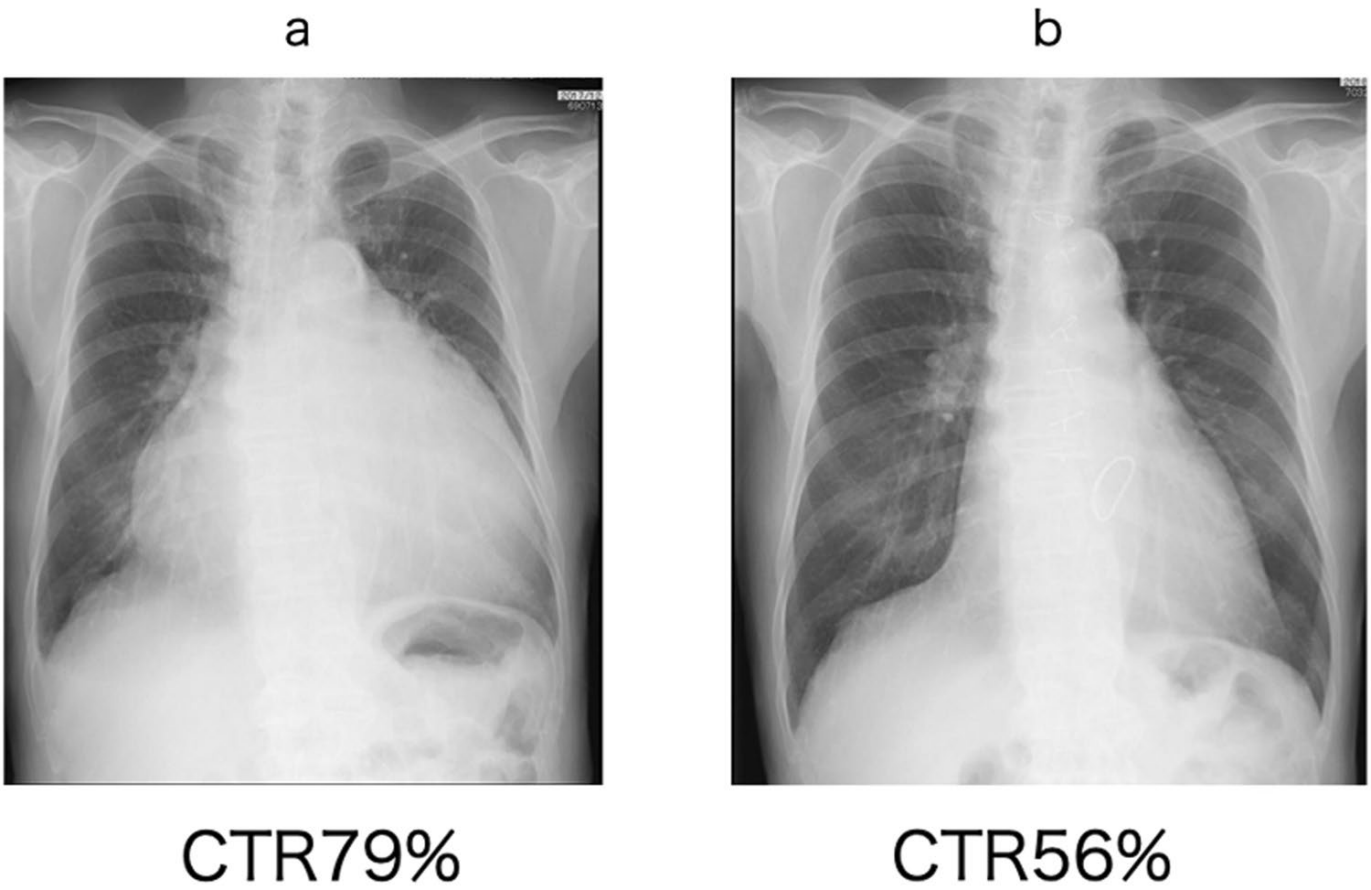
Chest radiographs (**a**) before surgery and (**b**) 3 months after an integrated operation involving mitral annuloplasty with patch augmentation, biatrial plication, and tricuspid annuloplasty. *CTR* cardiothoracic ratio

In most patients with atrial functional MR who undergo surgery, the f-wave on an electrocardiogram has disappeared if atrial fibrillation has been present for more than 10 years. Since both atria are markedly enlarged, the maze procedure is not indicated in most cases. However, as long as atrial fibrillation persists, expansion of the atrial cavity may potentially advance (Fig. 6). Although there is no clear standard, the left atrial plication procedure around the mitral valve annulus should be performed simultaneously in patients with a markedly enlarged atrium. We usually perform para-annular atrial plication in this situation [37]. Recently, Matsumori et al. reported no significant differences in mid-term morbidities between with and without left atrial plication in atrial functional MR cases [38]. Ohba et al. plicated the left atrium combined with mitral and tricuspid annuloplasty in all of their three patients [39]. They stated that para-annular plication is important for preventing the posterior mitral annulus from being pulled towards the posterior wall and returning it to its original position. We consider that the left atrial plication procedure around the mitral valve annulus should be performed simultaneously and the left atrial appendage must be closed. We also consider that these integrated surgical procedures will make the heart smaller and decrease the severity of heart failure (Fig. 7). Most patients with atrial functional MR have a long history of atrial fibrillation and the maze procedure is adaptable in only a few patients. Balogh et al. reported mitral valve repair in patients with HFpEF and 70% of patients received the maze procedure (mainly in the left atrium) [24]. However, these authors did not mention the conversion rate to sinus rhythm in their study.

## Catheter-based treatment for atrial functional MR

In a recent report, the catheter-based edge-to-edge method using a MitraClip™ (Abbott Vascular, Santa Clara, CA, USA) was applied to atrial functional MR [40]. However, this is not an essential treatment because it does not make the mitral annulus smaller. Whether this treatment should be applied for advanced cases with marked annular enlargement is unclear. We consider that the catheter-based edge-to-edge method might be useful as a palliative procedure for high-risk cases.

## Postoperative anticoagulation

When significant regurgitation exists in patients with atrial functional MR, the left atrium is always washed by the regurgitant jet. However, if the regurgitation disappears after valve repair or replacement, this rinsing action is lost, and blood flow stagnation occurs in the left atrium. Therefore, anticoagulant therapy should begin in the early postoperative period. Thrombi can also form in locations other than the left atrial appendage, such as the posterior wall of the left atrium. Warfarin is traditionally used for anticoagulant therapy; in some cases, however, the prothrombin time–international normalized ratio may take some time to reach the appropriate level. The use of direct oral anticoagulants is approved for patients with nonvalvular atrial fibrillation. Atrial fibrillation after mitral valve repair is considered a type of nonvalvular atrial fibrillation.

## Prognosis after surgery

Few articles have addressed the mid-term prognosis of surgical treatment. We retrospectively evaluated 45 patients with an ejection fraction of > 50% and found that the 5-year rates of freedom from postoperative valve-related re-administration and mortality were both 64% [22]. Abe et al. [4] reported that residual MR and TR after treatment for heart failure had a negative effect on the prognosis and that the prognosis for patients with both significant MR and TR was extremely poor. To the best of our knowledge, no studies have shown which treatment is better, medical therapy or surgery, and clinical trials comparing the two groups are required. Recently, Balogh et al. compared patients with atrial functional MR with HFpEF retrospectively with and without mitral valve repair [24]. The readmission rate for worsening HFpEF in the surgical group was 12%, which was significantly better than that in the conservative therapy group. These authors concluded that mitral valve repair provides long-term benefits in mortality and unplanned readmission. The significance of surgical treatment for atrial functional MR/TR should be proven in prospectively designed clinical trials.

## Conclusion

The pathology of atrial functional MR is completely different from that of conventional ventricular functional MR. Which patients with chronic atrial fibrillation will eventually develop atrial functional MR remains unclear. We emphasize that this pathology must be considered a dual-valve disease and that the conventional guidelines for single-valve disease should not be applied. More evidence is required regarding what surgical interventions should be performed and the optimal timing of their performance.
